# PARSID: a Python script for automatic analysis of local BLAST results for a rapid molecular taxonomic identification

**DOI:** 10.1186/s13104-024-06686-y

**Published:** 2024-01-24

**Authors:** Costanza Piccoli, Antonio Muñoz-Mérida, Angelica Crottini

**Affiliations:** 1 CIBIO, Centro de Investigação em Biodiversidade e Recursos Genéticos, InBIO Laboratório Associado, Universidade do Porto, Campus de Vairão, 4485-661 Vairão, Portugal; 2 Departamento de Biologia, Faculdade de Ciências, Universidade do Porto, Rua do Campo Alegre s/n, 4169– 007 Porto, Portugal; 3 BIOPOLIS Program in Genomics, Biodiversity and Land Planning, CIBIO, Campus de Vairão, 4485-661 Vairão, Portugal

**Keywords:** Blastn, DNA-barcoding, Molecular taxonomy

## Abstract

**Objective:**

A reliable taxonomic identification of species from molecular samples is the first step for many studies. For researchers unfamiliar with programming, running a BLAST analysis, filtering, and organizing results for hundreds of sequences through the BLAST web interface can be difficult. Additionally, sequences deposited in GenBank can have outdated taxonomic identification. The use of reliable Reference Sequences Library (RSL) containing accurate taxonomically-identified sequences facilitates this task. Pending the availability of a RSL with the user, we developed a tool that automates the molecular taxonomic identification of sequences.

**Results:**

We developed PARSID, a Python script running through the command-line that automates the routine workflow of blasting an input sequence file against the user’s RSL, and retrieves the matches with the highest percentage of identity in five steps. PARSID accepts cut-off parameters and supplementary information in a.*csv* file for filtering the results. The final output is visualized in a spreadsheet. We tested its functioning using 10 input sequences simulating different situations of the molecular taxonomic identification of sequences against an example RSL containing 25 sequences. Step-by-step instructions and test files are publicly available at https://github.com/kokinide/PARSID.git.

## Introduction

With the decrease of sequencing costs, the amount of genomic data generated and deposited in publicly available DNA databases (e.g. NCBI GenBank) has exponentially increased over the last decades. The progressive accumulation of sequences provides a solid comparison for newly-generated data. The BLAST (Basic Local Alignment Search Tool) algorithm [1] was implemented based on local similarity measures in sequence databases searches, leading to an increased interest towards using DNA fragments as taxonomic tools [e.g. 2, 3, 4, 5, 6]. The comparison process to identify unknown sequences relies on the availability of a comprehensive comparative molecular database and on recognized degrees of intraspecific and interspecific divergences in the genetic marker analysed (i.e. barcoding gap; [7]).

The correctness of the results obtained through BLAST searches against NCBI GenBank can be hampered by the accuracy of the taxonomic identification of the deposited material (e.g. mislabelling, submission errors, outdated taxonomic nomenclature, etc.), that can ultimately determine taxonomic misidentifications [8]. This problem can be solved by creating a local Reference Sequences Library for the group of interest using taxonomically accurate data. This step can also be performed in NCBI GenBank, but the processing and taxonomic identification of each individual sequence from large datasets remains time-consuming.

Here we present PARSID (PArser for Rapid Species IDentification), a Python script that obtains taxonomically-accurate molecular species identifications of multiple sequences of a selected marker, pending the availability of a RSL (Reference Sequences Library). Target users are researchers unfamiliar with programming. The aim of the script is automating the routine workflow that includes: (1) generating a local BLAST database from the user’s RSL; (2) performing the local BLAST; (3) parsing the BLAST result; (4) filtering and tagging dubious results; (5) organizing the final results in a spreadsheet.

## Main text

### Implementation

PARSID.py was developed in Python 3.8 (www.python.org), and its functioning requires the Biopython package (version 1.78; [9]; https://biopython.org), specifically the Bio.Blast, Bio.SearchIO, and Bio.SeqIO subpackages, and the XlsxWriter package (version 3.0.9; https://xlsxwriter.readthedocs.io/index.html). The source code and test files are available on GitHub [10] with detailed instructions. Before starting, BLAST+ [11] has to be locally installed to run BLAST from command line.

### Functioning

**Fig. 1:**
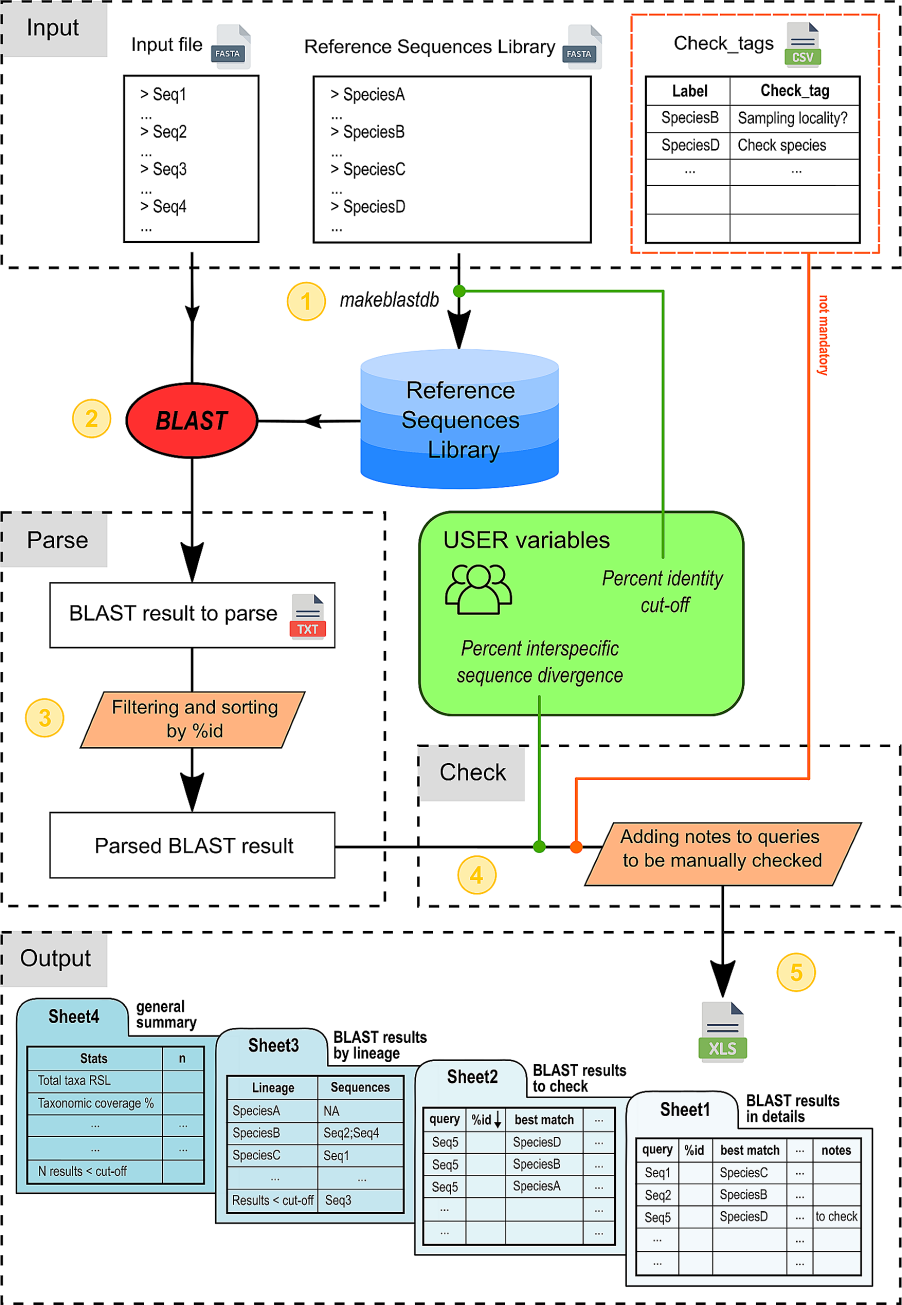
Workflow of PARSID.py in five steps. It performs in order: (1) creation of a custom reference database from a multi-FASTA sequence list, named Reference Sequences Library (RSL), (2) local BLAST of input file against the RSL, (3) BLAST result parsing and filtering, (4) parsed BLAST result checking through user’s parameter (*intersp_div*) and supplementary file (*Check_tags.csv*), and (5) organization of final results in spreadsheet format

PARSID.py automates the process of obtaining taxonomically-accurate molecular species identifications of multiple sequences from an input file in five steps and facilitates the visualization of the results (Fig. 1). The RSL is a customized multi-sequence database used to perform the local BLAST against the input sequence file. It contains single-marker reference sequences with unique identifiers for each described and candidate species of the analysed taxonomic group. While running the script, the user sets the cut-off percentage for sequence identity (user’s parameter: *cutoff_pident*), below which BLAST results are not saved, and the interspecific sequence divergence percentage (user’s parameter: *intersp_div*), which corresponds to the divergence threshold defining different taxa. Sequences with larger degrees of divergence in the BLAST analysis than the ones defined with the *intersp_div* are tagged for further checking. The user can provide supplementary tags for specific taxa through a.*csv* file that the script integrates in the final results file. The five main steps are explained below.

1. Generation of the local BLAST database using the customized RSL available with the user (Bio.Blast command *makeblastdb*). This step is the only one that runs from the shell. This step is executed prior using each different RSL.
2. Local BLAST of the input sequences and generation of the BLAST result file to parse. The output format is set on 12 standard fields in tabular format, adding the query length (*qlen*), the subject length (*slen*), the number of gaps (*gaps*), and the query coverage per subject (*qcovs*). The result file is saved in plain-text format that make it easier to consult and uses lower computer memory than the .*xml* format. It contains the hits with an identity percentage above the user’s *cutoff_pident*.
3. Parsing and filtering the local BLAST result. For each hit, the query coverage is calculated as:
  $$ qcov=\frac{(qend-qstart+1)}{qlen}*100$$
  Then the hits are filtered out by query coverage (< 75%), and by alignment length (< 90% of the subject length). These two parameters allow filtering those results that have high percentage of identity calculated on a shorter alignment length, in the case of a short query or short reference sequence. The results are then sorted by percentage of identity. The best-match for each query is retrieved.
4. Checking the parsed result by user’s parameter (*intersp_div*) and .*csv* supplementary file. The results that have a percentage of identity below the sequence similarity threshold, calculated as:
  $$ similarity threshold=100-intersp\_div$$
  or that have a query coverage below 75%, are tagged for further checking. The variability of the selected genetic marker is not always enough to enable accurate species identification for some taxa. In this case the user can integrate a supplementary file containing taxa-specific warnings so that the results of these specific sequences can be flagged and the metadata of these sequences (e.g. collection locality, voucher information, etc.) can be analysed to enhance identification.
5. Generation of the final output file. The output file can be visualized in a spreadsheet. The first sheet contains the list of the input sequences with the respective BLAST’s best-matches, including percentage of identity and query coverage. The results below the similarity threshold or with query coverage below 75% are color-coded for an easier visualization. The second sheet contains all the hits for those sequences tagged for further checking, either due to low percentage of identity or low query coverage. The third sheet contains the full list of lineages of the customized RSL with the respective sequences identifiers found in the input file, and also the list of sequences with a percentage of identity below the user’s *cutoff_pident*. The last sheet contains some general information, as the total number of lineages in the RSL, the taxonomic coverage of the RSL (i.e. the percentage of taxa of the RSL found in the input file), the total number of input sequences, the percentage of sequences processed (i.e. 100% if the script worked correctly), and the number of results below the user’s *cutoff_pident*.

### Testing

To simulate the molecular taxonomic identification of multiple sequences, we built a RSL using 25 sequences available in GenBank belonging to the frog family of Mantellidae of Madagascar for which the taxonomic identification was considered reliable (based on an expert-based approach). We tested the functioning of the script using an input file of 10 sequences available in GenBank (nine belonging to frogs of the family Mantellidae and one belonging to a species of the family Hyperoliidae). We set the *cutoff_pident* at 90% and the *intersp_div* at 3%. We included the optional.*csv* file to flag results of the genus “Mantella” or the RSL sequences of the herpetological collection of Museo Regionale di Scienze Naturali di Torino (“MRSN”). These test files are available in GitHub [10]. The input file included sequences with outdated taxonomic information, an outgroup (i.e. *Heterixalus*), and some sequences that needed further checking. The script successfully processed all sequences. The highest percentage of identity (*%id*) match was assigned to the nine mantellid sequences. Each result also included the following information to evaluate the query-subject alignment: *%qcov*, *qlen*, *slen*, *aln_len*, *evalue*, and *notes*. Of these nine sequences, three were highlighted in red and were tagged for further checking as the *%id* was below the similarity threshold initially set (i.e. 97%). Further results ordered by *%id* for these three sequences were listed in the second sheet of the result spreadsheet. Other sequences tagged for further checking contained information included in the.*csv* file. In the third sheet, results are organized by lineage and outgroups’ identifiers (i.e. sequences not belonging to Mantellidae were pooled together). In the fourth sheet, the script provided the taxonomic coverage in the RSL (28%), the number of taxa in the RSL without a sample (18 taxa), and the number of outgroups (1 sequence).

## Discussion

Molecular taxonomic identification is a fundamental step in species identification. PARSID.py facilitates the analysis of a large number of sequences, and allows the user to obtain filtered and organized results of molecular species identification in a short time. Despite lacking a graphical interface, the script is easy to run and the user is not required to have prior experience in programming. Indeed, the parameters to set for filtering the results are few and simple to introduce. By providing the script with supplementary taxa-specific check warnings, the user can customize the output following its needs and integrate additional information in the final results. Unlike the BLAST web interface, in which the hits are visualized for a single query at a time, PARSID.py’s final output shows the best-matches for all the queries and results are organized in a single spreadsheet, which is easy to edit and use for downstream analysis. For example, when analyzing a heterogeneous file of several unidentified sequences, it is useful to have all the analysed sequences sorted by lineage, to pool only the samples of a specific taxon.

### Limitations

This script enables the blasting, parsing, and filtering of sequences that otherwise can be performed by an expert bioinformatician through command-line shell. PARSID.py does not allow setting different *intersp_div* in the same analysis while analysing an input file containing sequences from different taxonomic groups, which would require having different threshold for molecular taxonomic identification. This is because the functionality of the script relies on defining the interspecific sequence divergence in distinguishing the taxa, while comparing the input file against the customized RSL. If different group of taxa show different thresholds of interspecific divergence, we recommend splitting the input file based on their higher taxonomic identification (e.g. genus, subfamily, family) and process each group separately applying different *intersp_div* threshold parameters. The reliability of the results depends on the quality of the user’s RSL. Computational time and memory usage depends on the dimension of the input file.

## Data Availability

The source code of this article is available in the GitHub repository, 10.5281/zenodo.8217541 (https://github.com/kokinide/PARSID.git) under the GNU General Public License v3.0.

## Abbreviations

BLAST: Basic Local Alignment Search Tool
PARSID: PArser for Rapid Species IDentification
RSL: Reference Sequences Library
