# Correction: PARSID: a Python script for automatic analysis of local BLAST results for a rapid molecular taxonomic identification

**DOI:** 10.1186/s13104-024-06771-2

**Published:** 2024-04-23

**Authors:** Costanza Piccoli, Antonio Muñoz-Mérida, Angelica Crottini

**Affiliations:** 1 CIBIO, Centro de Investigação em Bioaffiliationersidade e Recursos Genéticos, InBIO Laboratório Associado, Universidade do Porto, Campus de Vairão, 4485-661 Vairão, Portugal; 2 Departamento de Biologia, Faculdade de Ciências, Universidade do Porto, Rua do Campo Alegre s/n, 4169– 007 Porto, Portugal; 3 BIOPOLIS Program in Genomics, Bioaffiliationersity and Land Planning, CIBIO, Campus de Vairão, 4485-661 Vairão, Portugal

**Correction: BMC Research Notes (2024) 17:35**

10.1186/s13104-024-06686-y

The below funding information was omitted from the published article:

“Work supported by National Funds through FCT-Fundação para a Ciência e a Tecnologia in the scope of the project UIDP/50027/2020.”

The original article has been revised.

